# Navigation-Guided Transnasal Endoscopic Delineation of the Posterior Margin for Maxillary Sinus Cancers: A Preclinical Study

**DOI:** 10.3389/fonc.2021.747227

**Published:** 2021-11-11

**Authors:** Stefano Taboni, Marco Ferrari, Michael J. Daly, Harley H. L. Chan, Donovan Eu, Tommaso Gualtieri, Ashok R. Jethwa, Axel Sahovaler, Andrew Sewell, Wael Hasan, Ilyes Berania, Jimmy Qiu, John de Almeida, Piero Nicolai, Ralph W. Gilbert, Jonathan C. Irish

**Affiliations:** ^1^ Department of Otolaryngology—Head and Neck Surgery/Surgical Oncology, Princess Margaret Cancer Centre, University Health Network, Toronto, ON, Canada; ^2^ Section of Otorhinolaryngology—Head and Neck Surgery, Department of Neurosciences, University of Padua—“Azienda Ospedaliera di Padova”, Padua, Italy; ^3^ Guided Therapeutics (GTx) Program, Techna Institute, University Health Network, Toronto, ON, Canada; ^4^ University Health Network (UHN) Guided Therapeutics (GTx) Program International Scholar, Toronto, ON, Canada; ^5^ Artificial Intelligence in Medicine and Innovation in Clinical Research and Methodology (PhD Program), Department of Clinical and Experimental Sciences, University of Brescia, Brescia, Italy; ^6^ Technology for Health (PhD Program), Department of Information Engineering, University of Brescia, Brescia, Italy; ^7^ Unit of Otorhinolaryngology—Head and Neck Surgery, Department of Medical and Surgical Specialties, Radiologic Sciences, and Public Health, University of Brescia—“ASST Spedali Civili di Brescia”, Brescia, Italy; ^8^ Head & Neck Surgery, University College London Hospital, London, United Kingdom

**Keywords:** intraoperative navigation (NIV), 3D-virtual endoscopy, surgical margins, maxillary sinus cancers, transnasal endoscopic surgery

## Abstract

**Background:**

The resection of advanced maxillary sinus cancers can be challenging due to the anatomical proximity to surrounding critical anatomical structures. Transnasal endoscopy can effectively aid the delineation of the posterior margin of resection. Implementation with 3D-rendered surgical navigation with virtual endoscopy (3D-SNVE) may represent a step forward. This study aimed to demonstrate and quantify the benefits of this technology.

**Material and Method:**

Four maxillary tumor models with critical posterior extension were created in four artificial skulls (Sawbones^®^). Images were acquired with cone-beam computed tomography and the tumor and carotid were contoured. Eight head and neck surgeons were recruited for the simulations. Surgeons delineated the posterior margin of resection through a transnasal approach and avoided the carotid while establishing an adequate resection margin with respect to tumor extirpation. Three simulations were performed: 1) unguided: based on a pre-simulation study of cross-sectional imaging; 2) tumor-guided: guided by real-time tool tracking with 3D tumor and carotid rendering; 3) carotid-guided: tumor-guided with a 2-mm alert cloud surrounding the carotid. Distances of the planes from the carotid and tumor were classified as follows and the points of the plane were classified accordingly: “red”: through the carotid artery; “orange”: <2 mm from the carotid; “yellow”: >2 mm from the carotid and within the tumor or <5 mm from the tumor; “green”: >2 mm from the carotid and 5–10 mm from the tumor; and “blue”: >2 mm from the carotid and >10 mm from the tumor. The three techniques (unguided, tumor-guided, and carotid-guided) were compared.

**Results:**

3D-SNVE for the transnasal delineation of the posterior margin in maxillary tumor models significantly improved the rate of margin-negative clearance around the tumor and reduced damage to the carotid artery. “Green” cuts occurred in 52.4% in the unguided setting *versus* 62.1% and 64.9% in the tumor- and carotid-guided settings, respectively (*p* < 0.0001). “Red” cuts occurred 6.7% of the time in the unguided setting *versus* 0.9% and 1.0% in the tumor- and carotid-guided settings, respectively (*p* < 0.0001).

**Conclusions:**

This preclinical study has demonstrated that 3D-SNVE provides a substantial improvement of the posterior margin delineation in terms of safety and oncological adequacy. Translation into the clinical setting, with a meticulous assessment of the oncological outcomes, will be the proposed next step.

## Introduction

Resection of advanced maxillary sinus cancers can be particularly challenging due to the anatomical proximity to surrounding neural and vascular structures. This challenge creates a dilemma for surgical treatment as one is balancing between an adequate margin of resection and potential morbidity. Over the last three decades, the evolution of transnasal endoscopic surgery and improvements in adjuvant treatments have been considerably impacting the management of sinonasal cancer (1–13). Transnasal endoscopy can be considered the standard of treatment for many adequately selected nasoethmoidal malignancies; in addition, it can effectively aid the delineation of critical margins of resection even in the setting of open approaches for advanced sinonasal cancers (i.e., endoscopic-assisted maxillectomy and cranioendoscopic resection) (13, 14). With the era of endoscopic and minimal access surgical ablations, there has been increasing reliance on imaging for patient selection and for prediction of volume of ablation. The ability to increasingly employ intraoperative near real-time on-the-table surgical navigation (SN) to improve margin-negative resection is upon us.

With the advent of new technologies, particularly in the area of intraoperative imaging, the ability to increase the confidence and performance of margin-negative tumor resections while maximizing the preservation of normal anatomical structures is imminent. Specifically, determining the posterior margin (PM) of the resection during maxillectomy surgery is a challenge and has prompted researchers to propose solutions addressing this problem (13, 15–19). Correct delineation of the PM of a maxillectomy requires the surgeon to build a three-dimensional (3D) mental image of the tumor based on preoperative imaging. Even in the hands of experienced surgeons, this process can be difficult, and minor deviations in the position and orientation of the margin can significantly affect the cut trajectory with respect to the tumor and critical anatomical structures.

Since the early 1990s, SN has emerged as a useful aid and evolved parallel to transnasal endoscopic surgery, particularly with the intent to avoiding complications (20). SN in the craniomaxillofacial region has been proven to be useful in the assessment of the adequacy of reconstruction and for the planning of osteotomies during oncologic ablations (21–23). Moreover, SN has provided improved accuracy of craniomaxillofacial osteotomies (24, 25), and proportional improvement of clinical outcomes can be hypothesized based on preliminary experiences (26–29). Implementation of endoscopy with 3D-rendered SN with virtual endoscopy (3D-SNVE) may represent a significant step forward.

The aims of this preclinical study were to test and quantify the benefits provided by 3D-SNVE in terms of adequate delineation of the PM in models of advanced maxillary tumors that would require an open maxillectomy.

## Materials and Methods

### Tumor Model Preparation

**Figure 1:**
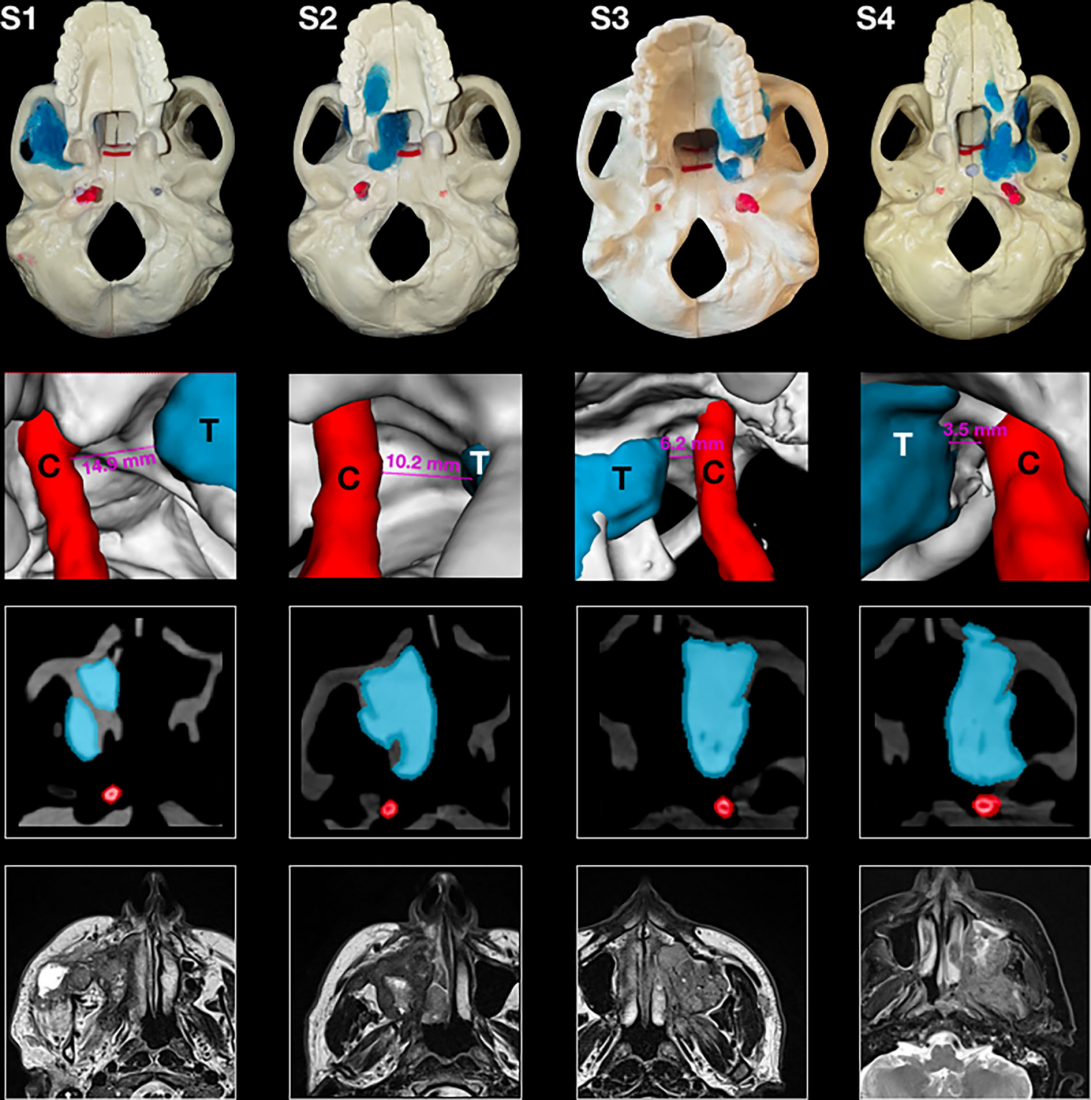
Panel with four phantoms, as seen from an inferior perspective (*superior row*); 3D rendering of the tumor and the carotid alongside the tumor–carotid distance for each model (*second row*); and appearance of tumors at the computed tomography imaging alongside contouring of the tumor and the carotid (*third row*); and preoperative magnetic resonance imaging (MRI) of four actual cases of maxillary cancers *(inferior row*).

**Figure 2:**
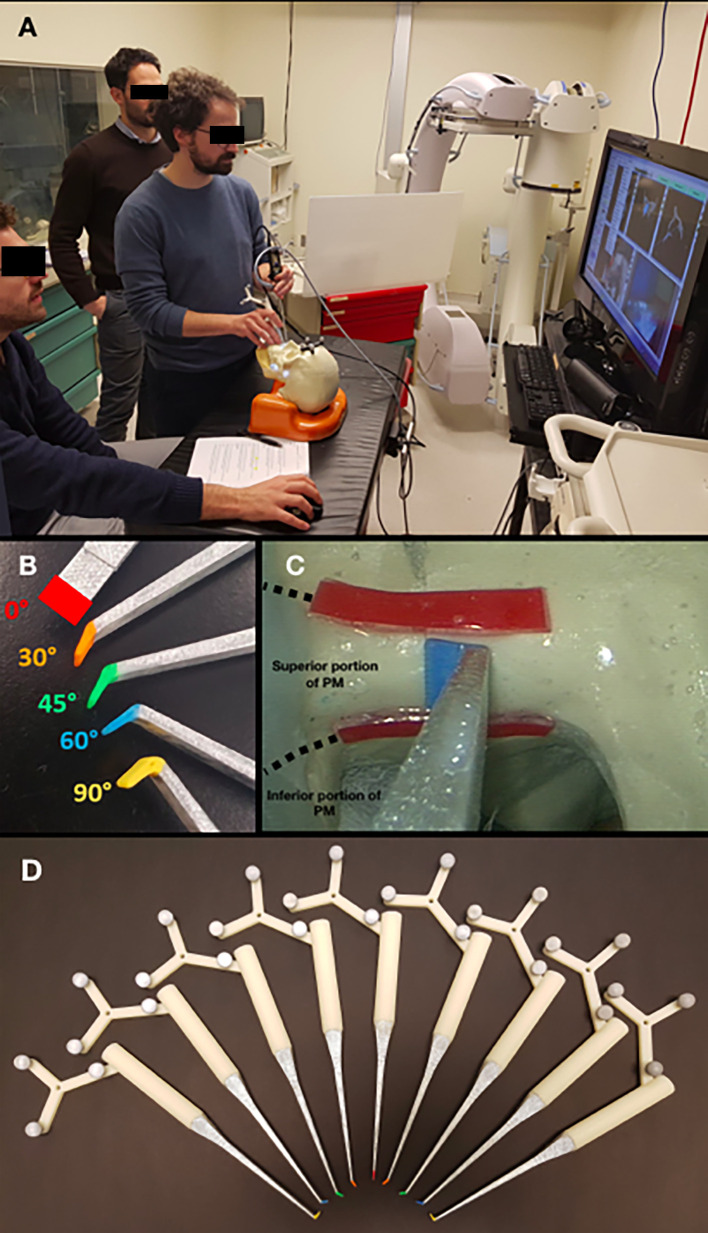
**(A)** Simulation setting. **(B)** Pointers with different types of angled tips. **(C)** Endoscopic view, with *red lines* indicating the superior and inferior potions of the posterior margin (PM); **(D)** Pointers with angled tips with different angles (0°, 30°, 45°, 60°, and 90°) and directions (right or left).

Four artificial skulls (Sawbones^®^, Vashon Island, WA, USA) and a moldable material (Play-Doh^®^, Hasbro^®^, Pawtucket, RI, USA) mixed with acrylic glue were employed to build four models (S1–S4) of locally advanced maxillary sinus tumors with varying degrees of posterior tumor extension. The degree of posterior extension in each model is described in terms of the involvement of anatomical spaces/structures and the closest distance from the internal carotid artery (ICA) (tumor–carotid distance, T–C distance), as follows: 1) invasion of the pterygopalatine fossa (PPF) (T–C distance = 14.9 mm, model S1); 2) invasion of the medial pterygoid plate, pterygoid fossa, and base of the pterygoid process (T–C distance = 10.2 mm, model S2); 3) complete invasion of the pterygoid process (T–C distance = 6.2 mm, model S3); and 4) invasion of the anterior foramen lacerum and upper parapharyngeal space (T–C distance = 3.5 mm, model S4) (**Figure 1**). Each tumor model was created based on actual cases of maxillary cancers treated between January 2016 and December 2018 in the Unit of Otorhinolaryngology – Head and Neck Surgery of the University of Brescia (Brescia, Italy). The tumor models were based on preoperative magnetic resonance imaging (MRI) (**Figure 1**). Soft tissues in the models were simulated using medical gauzes to restrict tumor visualization to only the endoscopic and transoral views (i.e., simulating tumors ulcerating into the sinonasal and/or oral cavity). The anterior third of the nasal septum was simulated with a 3-mm slice of silicon, fixed orthotopically to the skull with acrylic glue. As a result of silicon elasticity, the anterior nasal septum could be partially tilted and displaced with the scope and instruments during simulations. The ICAs in the models were created from an angio-CT that was done in a neurological workup for an anonymized patient and were semi-automatically contoured through Mimics^®^ (Materialise, Leuven, Belgium). Firstly, a global threshold was applied to provide a quick gross segmentation, and then manual refinement was used to smooth the segmentation. Respective stereolithography (STL) files were generated and ICAs were 3D printed (3D Printer Dimension 1200es System; Stratasys, Eden Prairie, MN, USA) and painted with red dye mixed with iodine solution for CT contrast (Omnipaque; GE Healthcare, Chicago, IL, USA). A carotid canal was manually created in the base of the artificial skulls, and each ICA was fixed in the anatomical situation. The area for simulation of transnasal PM delineation was marked by horizontal lines in the phantoms and further classified into a superior and an inferior part based on the plane passing through the inferior aspect of the nasopharyngeal vault (**Figure 2C**). 

### Surgical Pointer Tool Preparation

Custom surgical pointers were designed using Autodesk Fusion 360 software (San Rafael, CA, USA) and 3D printed on a Dimension 1200es System (Stratasys, Eden Prairie, MN, USA). Surgeons participating in the simulations were provided with color-coded pointers with different angulations (0°, 30°, 45°, 60°, and 90°) (**Figures 2A, B, D**). Each pointer was meant to simulate the trajectory of delineation of the PM of resection, so that the surgeon could select which trajectory best represented the way he/she would have set the PM of resection from a transnasal perspective.

### Image Acquisition and Tumor Contouring

**Figure 3:**
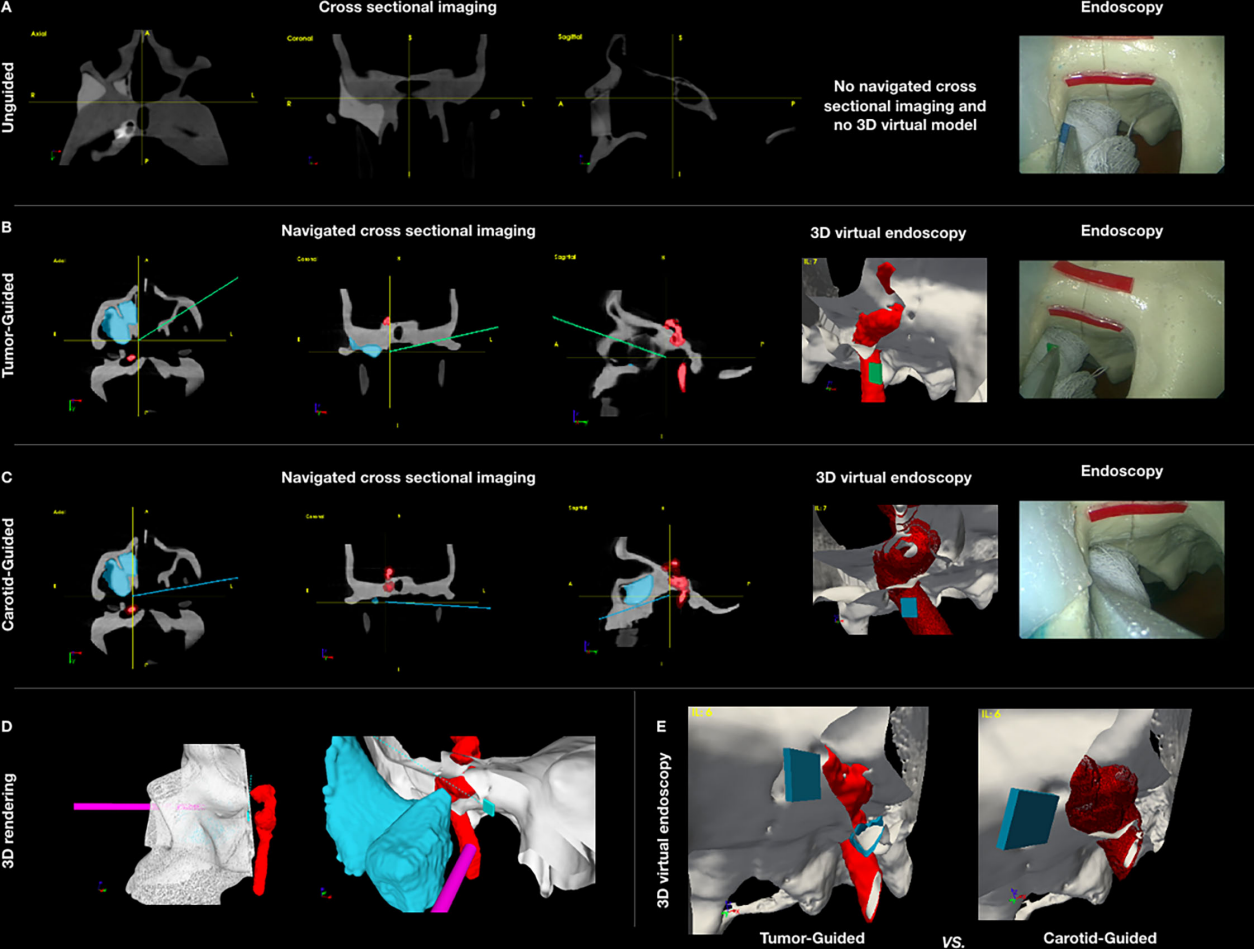
**(A–C)** Panel showing the appearance of the different settings of data acquisition: unguided simulations with cross-sectional imaging pre-simulation analysis **(A)**, tumor-guided **(B)**, and carotid-guided **(C)** simulations with real-time surgical navigation indicating the position of the instrument and the posterior margin delineation. **(D)** Pictures showing the appearance of the 3D rendering of the skull with the positions of the scope and pointer. Virtual margin delineation, simulating the cut of 3D objects (skull, tumor, and carotid). **(E)** Comparison of the 3D virtual endoscopy appearance in the tumor-guided and carotid-guided settings.

Three-dimensional images of each skull model were acquired using a prototype cone-beam computed tomography (CBCT) imaging system on a mobile C-arm (30, 31). The mentioned flat-panel imaging system was validated for guidance of head and neck procedures involving significant bone resection and/or complex anatomical reconstruction (32). In this study, 3D volumes (256 × 256 × 192) covered a field of view of 20 × 20 × 15 cm^3^ using isotropic 0.8-mm 3D voxels. On CBCT imaging, the tumor and carotid models were clearly distinguishable from the artificial bone, as they showed a much higher X-ray attenuation (**Figures 1** and **3**). Contouring of the tumors and the ICAs was obtained semi-automatically using a two-step process within the NIRFAST-Slicer software (33). Firstly, a global threshold was applied to provide a quick, coarse segmentation, and then manual refinement was used to smooth the segmentation. To visualize a virtual surgical margin around the ICA (**Figure 3**), a semi-transparent wireframe was generated at 2 mm from the vessel surface using volumetric image dilation processing in MATLAB software (MathWorks, Natick, MA, USA).

### Navigation System

CBCT images were displayed within an in-house navigation software package (GTx-Eyes) based on the open-source Image-Guided Surgery Toolkit (34, 35). Tumor and margin segmentations were superimposed on three-planar views and separately as 3D surface renderings. Surgical tool tracking in this study was provided by a stereoscopic infrared camera (Polaris Spectra, NDI, Waterloo, Canada). Image-to-tracker registration was obtained by paired-point matching of predrilled divots by means of a tracked pointer. A small four-sphere reference tool (NDI, Waterloo, Canada) was anchored to the skull throughout the registration and simulations. A registration error of 1 mm or less was considered acceptable for the navigation experiments. A four-sphere reference (Medtronic, Jacksonville, FL, USA) was secured to each 3D printed tool (surgical pointer) and to a Storz^®^ endoscope (Karl Storz Group, Tuttlingen, Germany), which was then calibrated using a custom calibration jig. Angled pointer navigation was implemented using software features for virtual planar tool clipping (e.g., osteotome or saw) and colored accuracy indicators for distance, pitch, and roll developed previously for orthopedic oncology applications (36) and subsequently applied to open head and neck procedures (24, 25, 37). In this study, for transnasal simulations, the 3D rendering of the virtual endoscopic view could be freely rotated and the skull rendering clipped along the virtual cutting plane during the transnasal delineation of the PM (**Figure 3**).

### Surgical Simulation

Surgeons from the Department of Otolaryngology – Head and Neck Surgery of the University Health Network (Toronto, ON, Canada) and from the Unit of Otorhinolaryngology – Head and Neck Surgery of the University of Brescia (Brescia, Italy) were recruited for the simulations. Each surgeon received a brief explanation of the steps of the simulation and of the subsequent analysis methods. The surgical task was to choose among pointers with different angulations (0°, 30°, 45°, 60°, and 90°) and position the selected pointer under transnasal endoscopy guidance within the delineated areas (i.e., superior and inferior parts of the PM of resection) to provide a clear margin from the tumor posterior surface while avoiding intersection with the ipsilateral ICA. No physical cuts were performed to allow reuse of the models; rather, the pointer position and orientation were recorded when the surgeon gave vocal confirmation of his/her proposed delineation of the margin, and the analysis was performed on the virtual trajectory. Surgeons were asked to define the superior and posterior parts of the PM with two endoscopes (0° and 45°), first using only the surgical corridor of the ipsilateral nasal cavity and then through either a bilateral (i.e., with the scope through one nostril and pointer through the other) or a contralateral approach (i.e., with both scope and the pointer through the contralateral nostril). Surgeons were required to perform the PM delineation in three settings—1) unguided, 2) tumor-guided, and 3) carotid-guided—as shown in **Figure 3**. In the unguided simulation, the surgeons could only view the cross-sectional images (i.e., axial, sagittal, and coronal) prior to starting transnasal endoscopy, with no access to the real-time navigation system or the 3D tumor/margin renderings. In the tumor-guided simulation, virtual angled pointers were guided using real-time tool tracking and the 3D tumor and carotid segmentation (**Figure 3**). Finally, in the carotid-guided simulation, a 2-mm alert cloud surrounding the carotid was added to the tumor-guided setting; in this setting, a sonic alarm reproducing the arterial flow sound at Doppler examination was sounded when the trajectory of the PM definition was through the proximity alert zone (38), and a beeping sonic alarm was activated when the trajectory of the PM definition was through the ICA (**Figure 3**).

To avoid recall bias, the phantoms were randomized for each surgeon and the sequence of the phantoms was arranged such that the guided and unguided simulations were never performed at close intervals. The rationale for this was based on the belief that guided simulations could have enhanced adequate pointer orientation in a subsequent unguided task. Written informed consent was obtained from the individuals for the publication of any potentially identifiable images or data included in this article.

### Virtual Cutting Plane Analysis

**Figure 4:**
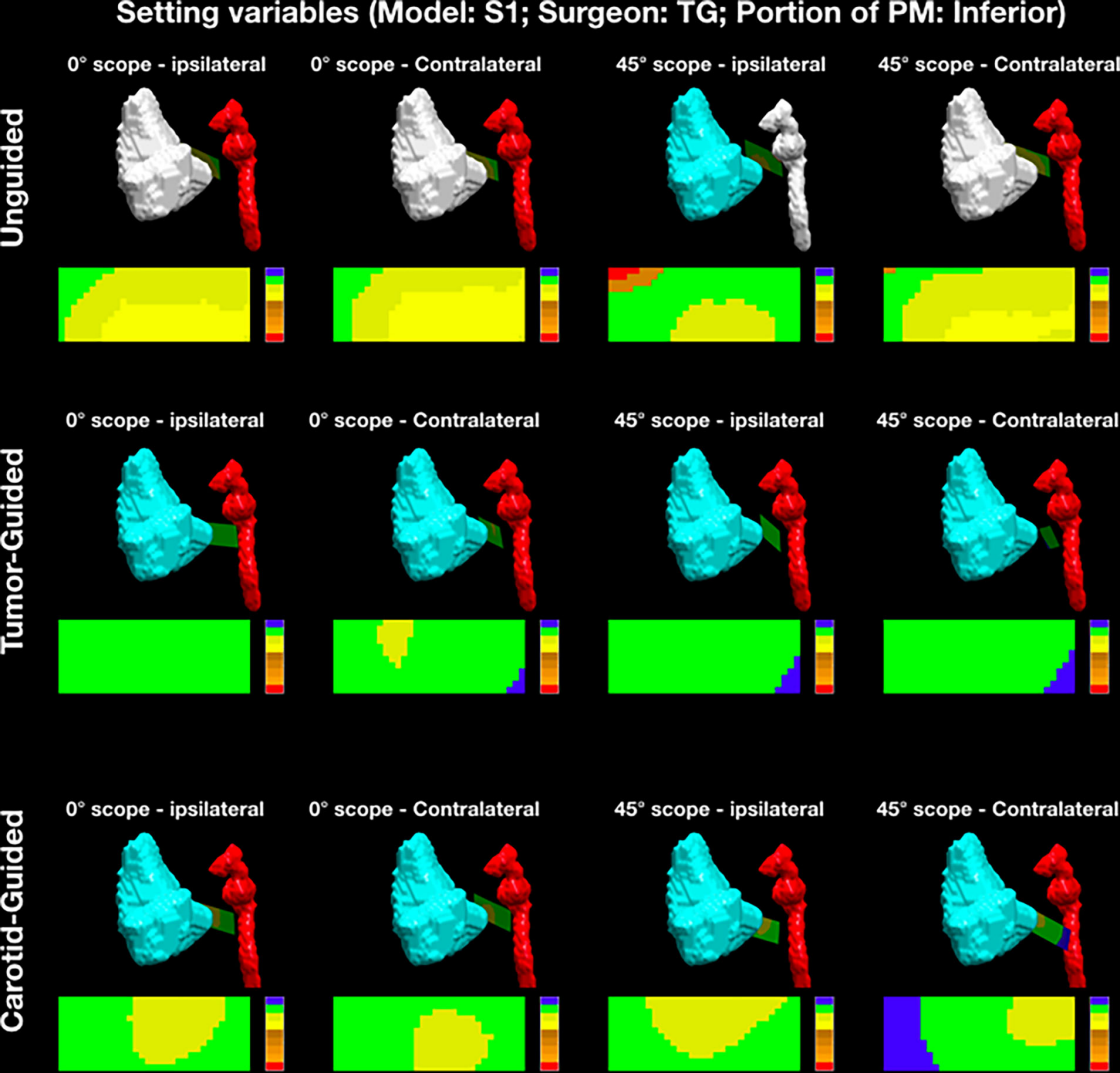
Example of the analysis of posterior margin delineation. Each *point* of the isolated area was classified as follows: “red” (*R*), into the internal carotid artery (ICA); “orange 1” (*O1*), <2 mm from the ICA and into the tumor; “orange 2” (*O2*), <2 mm from the ICA and <5 mm from the tumor; “orange 3” (*O3*), <2 mm from the ICA and 5–10 mm from the tumor; “orange 4” (*O4*), <2 mm from the ICA and >10 mm from the tumor; “yellow 1” (*Y1*), >2 mm from the ICA and into the tumor; “yellow 2” (*Y2*), >2 mm from the ICA and <5 mm from the tumor; “green” (*G*), >2 mm from the ICA and 5–10 mm from the tumor; and “blue” (*B*), >2 mm from the ICA and >10 mm from the tumor. *PM*, posterior margin.

Analysis of the cutting planes was performed by means of MATLAB software (MathWorks, Natick, MA, USA). An area of 30-mm length along the longitudinal axis of the cut and 11-mm width (5.5 mm on both sides with respect to the longitudinal axis) was isolated from each plane starting from the pointer tip. The minimal distance with respect to the tumor and ICA surfaces was calculated for each point making up the isolated area and reproduced as a distribution of distances shown as a 30 × 11-mm^2^ (length × width) color-scaled image (**Figure 4**). The cutting plane was deemed to be “intratumoral” when the distance from the cutting plane to the tumor was ≤0 mm and “adequate” when it was >0 mm. If the cutting plane was ≤0 mm to the ICA wall, the ICA was considered “damaged”, while a 0- to 2-mm margin to the ICA was deemed to be a “danger zone”. An “adequate” distance was defined as >2 mm. Each point of the isolated area was classified as follows: “red” (R), into the ICA; “orange 1” (O1), <2 mm from the ICA and into the tumor; “orange 2” (O2), <2 mm from the ICA and <5 mm from the tumor; “orange 3” (O3), <2 mm from the ICA and 5–10 mm from the tumor; “orange 4” (O4), <2 mm from the ICA and >10 mm from the tumor; “yellow 1” (Y1), >2 mm from the ICA and into the tumor; “yellow 2” (Y2), >2 mm from the ICA and <5 mm from the tumor; “green” (G), >2 mm from the ICA and 5–10 mm from the tumor; and “blue” (B), >2 mm from the ICA and >10 mm from the tumor. Each isolated area was described as a distribution among the above-mentioned categories.

### Surgeon Questionnaire

At the end of the simulations, each surgeon was asked to complete a validated questionnaire (38) (**Table 1**) in order to quantify opinions about the gain in terms of speed, accuracy, and self-confidence using tool tracking and the proximity alerts alongside the readiness for clinical translation of the technology.

**Table 1:** Questionnaire answers and surgeons’ responses.

Statements for questionnaire (38)	Median (IQR)^a^
I felt faster to perform surgery when aided by the virtual view.	6.0 (6.0–6.8)
The system appeared to be sufficiently accurate for its intended use.	6.0 (6.0–6.0)
The dynamic tool tracking allowed me to quickly assess my proximity to critical structures without significantly interrupting dissection.	6.0 (6.0–6.8)
Proximity alerts increased my confidence during ablation close to critical structures.	6.0 (6.0–6.8)
The current technology is ready for clinical trial without significant changes.	5.5 (4.3–6.0)

IQR, interquartile range.

a Based on a seven-point Likert scale (7 = strongly agree; 1 = strongly disagree).

### Statistical Analysis

Statistical analysis was run through XLSTAT^®^ (Addinsoft^®^, Long Island, NY, USA). Simulations were grouped into three categories: unguided, tumor-guided, and carotid-guided. These three groups were compared in terms of the distance distributions through a bilateral Kruskal–Wallis test and the Steel–Dwass–Critchlow–Fligner *post-hoc* test. The rates of intratumoral and intra-ICA virtual cuts among the three groups of simulations were assessed with Fisher’s exact test. Intraindividual differences in terms of percentage of adequate distance (G area) between the tumor/carotid-guided and unguided groups of simulations were calculated and considered as the “gain” provided by 3D-SNVE. The level of significance was set at 0.05 for all statistical tests.

## Results

Eight head and neck surgeons with heterogeneous experience (ranging from 3 to 13 years of experience) in oncologic endoscopic resections participated in the study. Five surgeons completed head and neck fellowship training, while three were attending a residency training program at the time of simulations. Overall, 612 PM transnasal delineations were simulated, namely, 204 per group (i.e., unguided, tumor-guided, carotid-guided). The registration error was <1 mm in all simulations. Surgeons chose to use the 0°, 30°, 45°, 60°, and 90° pointers in 0 (0%), 61 (11%), 99 (18%), 246 (45%), and 138 (25%) transnasal simulations, respectively. Surgeons indicated that the surgical exposure was not adequate in 68 (11%) of the simulations, of which 61 (90%) were through a transnasal ipsilateral approach, and no plane trajectories were recorded in these cases.

The virtual delineation of the PM of resection in maxillary tumor models transgressed the tumor in 47 (25.4%), 7 (4.0%), and 4 (2.2%) cases in the unguided, tumor-guided, and carotid-guided procedures, respectively (*p* < 0.0001). The virtual margin delineation was more than 2 mm to the ICA in 80 (43.2%), 104 (59.4%), and 111 (60.3%) cases in the unguided, tumor-guided, and carotid-guided procedures, respectively (*p* < 0.0001) and involved the ICA in 79 (42.7%), 30 (17.1%), and 25 (13.6%) cases in the unguided, tumor-guided, and carotid-guided procedures, respectively (*p* < 0.0001).

Simulation tumor model S1 had a significantly lower rate of points falling into the carotid (at least one point into the carotid in 6% of simulations) and in the 2-mm carotid alert zone (at least one point into the alert zone in 7% of simulations) when compared to phantoms S2, S3, and S4 (32%, 30%, and 18% of intra-carotid simulations and 32%, 36%, and 35% of intra-alert zone simulations, respectively; *p* < 0.0001 for both comparisons). The rate of clear margin (i.e., margin not crossing the tumor) was not significantly different among the four phantoms (92%, 86%, 90%, and 87% for S1, S2, S3, and S4, respectively; *p* = 0.33).

The percentage of points falling within the tumor volume was significantly higher in the group of unguided simulations compared to the tumor- and carotid-guided ones (*p* < 0.0001) (**Table 2**). In a bivariate analysis, the guidance proved to be associated with a higher rate of clear margin (*p* < 0.0001) and a lower rate of carotid damage (*p* < 0.0001), independently of the increasing difficulty of the tumor–carotid model.

**Table 2:** Average percentage of points of the virtual margin delineation in each category of the “color code” according to the guidance setting.

Color code	Description	% of Cutting planes			*p*-value
		Unguided	Tumor-guided	Carotid-guided	
Red	Into the carotid	6.7	0.9	1.0	*p* < 0.0001
Orange 1	<2 mm carotid, into the tumor	0.0	0.0	0.0	NS
Orange 2	<2 mm carotid, <5 mm tumor	0.3	0.2	0.3	NS
Orange 3	<2 mm carotid, 5–10 mm tumor	4.3	2.8	2.1	*p* < 0.0001
Orange 4	<2 mm carotid, >10 mm tumor	1.6	0.2	0.4	*p* < 0.0001
Yellow 1	>2 mm carotid, into the tumor	3.6	0.4	0.2	*p* < 0.0001
Yellow 2	>2 mm carotid, <5 mm tumor	19.1	23.8	23.5	*p* = 0.041
Green	>2 mm carotid, 5–10 mm tumor	52.4	62.1	64.9	*p* < 0.0001
Blue	>2 mm carotid, >10 mm tumor	12.1	9.5	7.5	NS

NS, not significant.

3D-SNVE significantly improved the rate of identification of an adequate plane of dissection while reducing the risk of carotid damage: the percentage of “red” points was significantly lower in the two guided groups with respect to the unguided group (*p* < 0.0001) (**Table 2**), and the percentage of points with an adequate distance from the carotid and the tumor simultaneously (i.e., “green” points) was significantly higher in the guided groups when compared to the unguided group (*p* < 0.0001) (**Table 2**).

The gain of margin delineation provided by 3D-SNVE (considering both tumor- and carotid-guided settings) was, on average, 24.2% (ranging from 0.0% to 33.3%, when analyzing single-surgeon results) in terms of obtaining clear margins and 25.7% (ranging from 1.8% to 59.6%, when analyzing single-surgeon results) in terms of avoiding carotid damage. The heterogeneity of training and experience resulted in a significant variability of the rates of intratumoral unguided cuts between surgeons (average value = 9.6%, range = 0.0%–16.7%, *p* = 0.039), but the gain in the adequacy of margin delineation provided by 3D-SNVE was statistically independent of the operator (*p* = 0.202).

### Surgeon Preference

All surgeons preferred using a bilateral transseptal approach to have better vision and working volume in all models. Surgeons felt more self-confident using the 0° and 45° scopes in 68% and 32% of unguided simulations and in 46% and 54% of guided simulations, respectively. When using 3D-SNVE, surgeons preferred the carotid-guided setting in 61% of the simulations and the tumor-guided in 39%.

### Questionnaire Score

The seven-point Likert scale questionnaire statements and median (interquartile range, IQR) responses are shown in **Table 1**. No subject strongly disagreed (scores 1–2) with any of the statements. Only one gave a negative response (score of 3) to question 1. One gave a negative response (score of 3) and two gave a neutral response (score of 4) to question 7. There was universal agreement (scores 5–7) for all other questions, with uniform responses across the subjects.

## Discussion

The present preclinical study demonstrates the beneficial role of 3D-SNVE in PM delineation and ICA preservation in ablative surgery for advanced maxillary tumors.

The frequency of “positive” margins decreased from 27% to 3% when the surgeon used navigation during the simulation, and carotid damage decreased from 41% to 15%. Since margin control still represents a challenging goal in the surgical management of such cancers, implementation of 3D-SNVE into surgical practice is a promising strategy for the future. Furthermore, the possibility of adding 3D rendering of the critical structures on virtual views and cross-sectional imaging with associated sound alerts may increase the confidence of the surgeon during the procedure and help avoid life-threatening complications.

While surgery combined with neoadjuvant and adjuvant radiation and chemotherapy has improved the overall outcomes of advanced sinonasal cancers, surgery still remains the principal modality of treatment (39–41). Clear-margin resection has been proven to significantly impact patient prognosis and can be considered the most important surgeon-controllable variable (13, 42–45). Endoscopic surgery has been shown to improve the surgical precision and to reduce the morbidity of certain procedures. The benefits of guiding margin delineation in open maxillectomies through an endoscopic transnasal approach was demonstrated by Deganello et al. (13), who reported this technique as facilitating the detachment of the maxilla from the skull base and allowing for a more precise delineation of the posterior and medial margins of resection. This endoscopic technique was used to treat 79 advanced tumors involving the maxilla with a low rate of microscopic involvement of the PM (3.8%) (13). The authors classified posterior endoscopic resection into three types according to the anatomical structures progressively involved and found that, even in the most complex scenario (i.e., type 3 resection), the rate of free PM was remarkably high (87.5%) (13).

In previous clinical studies by Catanzaro et al. (26) and Tarsitano et al. (29), 3D navigation was helpful in achieving a significantly higher rate of clear deep margin when implemented to the standard procedure for advanced maxillary, oral, or orbital cancers (i.e., ablation followed by mapping of the surgical bed with frozen section biopsies). More recently, in studying maxillectomy surgery, Ricotta et al. (46) confirmed that the rate of overall positive margins was higher in the control group (10 patients) compared to that in a group of 18 patients operated on with SN.

A preclinical study by Ferrari et al. (24) was performed using a previous version of the same in-house navigation system employed here. That study evaluated cutting planes for osteotomies in open surgery of sinonasal advanced cancers and demonstrated a substantial benefit in the delineation of the virtual osteotomies both for novel and experienced surgeons.

The present study adds to this previous work by testing the navigation system in a more complex setting, with critical anatomical structures close to the tumor. In addition to the complexity of the tumor–vessel model, further development of real-time tool tracking with 3D virtual endoscopy for angled endoscopes allowed visual overlay of the structures beyond the confines of the nasal wall and further allowed clipping of the endoscopic 3D rendering along the angled pointer trajectory.

The surgical treatment of maxillary tumors requires accurate delineation of the posterior boundary of the resection in a very complex area with surrounding critical anatomical structures. The surgeon needs to base the ablation planning on a mental representation of the tumor and surrounding structures, relying upon specific anatomical landmarks identified throughout the dissection, and this task becomes particularly challenging at the PM owing to poor visualization and maneuverability. Furthermore, cancers frequently have an irregular shape and have complex patterns of invasion into neighboring structures (47). The use of 3D navigation provides the surgeon with a real-time direct visualization of the tumor and the adjacent critical structures and facilitates positioning and orienting the margin with respect to the tumor and critical structures. The clinical translation of this navigation approach may help in achieving a balance between the adequacy of the oncological resection and preservation of the uninvolved surrounding anatomical structures. This benefit of the navigation has already been demonstrated in the field of pelvic tumor resection (36).

In our preclinical study, a significant improvement in the virtual delineation of maxillectomy PM with high rates of complete and ICA-sparing virtual resection was demonstrated when 3D-SNVE was employed. The benefit of margin delineation guided by SN in terms of oncologic adequacy and critical structure preservation was remarkable (average gain of 24.2% in obtaining clear margins and 25.7% in avoiding carotid damage). Despite the heterogeneity of training and experience, which resulted in a significant variability of the rates of intratumoral unguided cuts (*p* = 0.039), the gain in the adequacy of margin delineation when relying on 3D-SNVE was statistically independent of the surgeon (*p* = 0.202). This result suggests that SN could be beneficial both for expert and novice surgeons. The most reasonable explanation is that the 3D visualization of the tumor facilitates margin delineation, thus partially compensating for lack of experience in 3D mental representation of the tumor position and boundaries. In addition, with more extensive use of this technology, a learning curve with further improvements in surgical precision and time required can be expected, as already observed in other studies focusing on SN in the sinonasal area (21).

Margins were not classified into either adequate or close for two main reasons: 1) the definition of a “negative”, “close”, or a “positive” margin is not clear for sinonasal cancer resections, and 2) a complete resection with a 5-mm or wider margin is hardly ever achievable in sinonasal cancers. In the present study, we created phantoms with tumor models mimicking real cases with a very critical posterior extension, in which the minimal distance between the ICA and the tumor was 8.7 mm, on average (median = 8.2 mm, range = 3.5–14.9 mm).

The preclinical nature of the present study represents its main limitation, as the results could be potentially biased by the “ideal” conditions of the laboratory setting. Therefore, the benefits conferred by 3D-SNVE should be interpreted cautiously. However, the preliminary clinical data published in the literature are in agreement with the conclusion of our experiment (26, 28, 29, 37, 46). Translation of 3D-SNVE into clinical research should be the next step in order to test the potential benefits on patient outcomes with application in live surgery and in the environment of an operating room. Another limitation was that an arbitrary area of 30 × 11 mm was chosen to simulate the endoscopic PM delineation; in real surgery, the margin shape would result more irregular and variably sized.

The authors acknowledge that repeating simulations with the same technology, even if with different guidance modalities, may have caused a “learning effect”. Future studies will also investigate the benefits of navigation across a wider range of experience levels, including senior staff.

## Conclusion

This preclinical study has demonstrated the substantial benefit of 3D-SNVE for PM definition in advanced maxillary tumors. This technology is expected to improve resection margins and potentially reduce critical structure injury, thus optimizing the oncological adequacy and overall safety of the resection simultaneously. Translation into the clinical setting, with a thoughtful analysis of oncological outcomes, is the proposed next step.

## Data Availability Statement

The raw data supporting the conclusions of this article will be made available by the authors, without undue reservation.

## Author Contributions

ST, MF, MD, JI, RG, and PN contributed to the study concepts. MF, MD, ST, and HC designed the study. ST, MD, MF, TG, DE, AJ, ASa, WH, ASe, IB, and JD helped with data acquisition. All authors analyzed and interpreted the data. ST, MF, and MD performed the statistical analysis. ST, MF, and MD prepared the manuscript. ST, MF, MD, JI, RG, and PN edited the manuscript. All authors contributed to the article and approved the submitted version.

## Funding

Funding was provided by the Princess Margaret Cancer Foundation (Toronto, Canada), including the Kevin and Sandra Sullivan Chair in Surgical Oncology, the Myron and Berna Garron Fund, the Strobele Family Fund, and the RACH Funds.

## Conflict of Interest

The authors declare that the research was conducted in the absence of any commercial or financial relationships that could be construed as a potential conflict of interest.

## Publisher’s Note

All claims expressed in this article are solely those of the authors and do not necessarily represent those of their affiliated organizations, or those of the publisher, the editors and the reviewers. Any product that may be evaluated in this article, or claim that may be made by its manufacturer, is not guaranteed or endorsed by the publisher.
